# Systematic review of treatment intensification using novel agents for chemoradiotherapy in rectal cancer

**DOI:** 10.1002/bjs.10993

**Published:** 2018-10-12

**Authors:** R. Clifford, N. Govindarajah, J. L. Parsons, S. Gollins, N. P. West, D. Vimalachandran

**Affiliations:** ^1^ Institute of Cancer Medicine, University of Liverpool Liverpool UK; ^2^ North Wales Cancer Treatment Centre, Glan Clwyd Hospital Bodelwyddan UK; ^3^ Leeds Institute of Cancer and Pathology, University of Leeds Leeds UK; ^4^ Department of Colorectal Surgery Countess of Chester NHS Foundation Trust Chester UK

## Abstract

**Background:**

With the well established shift to neoadjuvant treatment for locally advanced rectal cancer, there is increasing focus on the use of radiosensitizers to improve the efficacy and tolerability of radiotherapy. There currently exist few randomized data exploring novel radiosensitizers to improve response and it is unclear what the clinical endpoints of such trials should be.

**Methods:**

A qualitative systematic review was performed according to the PRISMA guidelines using preset search criteria across the PubMed, Cochrane and Scopus databases from 1990 to 2017. Additional results were generated from the reference lists of included papers.

**Results:**

A total of 123 papers were identified, of which 37 were included; a further 60 articles were obtained from additional referencing to give a total of 97 articles. Neoadjuvant radiosensitization for locally advanced rectal cancer using fluoropyrimidine‐based chemotherapy remains the standard of treatment. The oral derivative capecitabine has practical advantages over 5‐fluorouracil, with equal efficacy, but the addition of a second chemotherapeutic agent has yet to show a consistent significant efficacy benefit in randomized clinical assessment. Preclinical and early‐phase trials are progressing with promising novel agents, such as small molecular inhibitors and nanoparticles.

**Conclusion:**

Despite extensive research and promising preclinical studies, a definite further agent in addition to fluoropyrimidines that consistently improves response rate has yet to be found.

## Abstract 2

Promising agents coming

## Introduction

Rectal cancer treatment has continued to improve in recent years as a result of optimized surgical technique, advances in staging, pathological quality control and multidisciplinary management. Neoadjuvant chemoradiotherapy (CRT) is considered the standard of care for locally advanced rectal cancer (LARC). It is well recognized that the response to neoadjuvant CRT is both variable and unpredictable for the individual patient, and techniques to risk‐stratify patients and predict response are an expanding area of research. Favourable responses to CRT are independently associated with conferring a long‐term survival advantage to patients who undergo resection, and in more recent years the possibility of deferral of surgery and organ preservation has also been raised^1^.

A complete response to CRT may be classified as either a clinical complete response (cCR) or a pathological complete response (pCR). Although the two terms are often used interchangeably, these responses are assessed differently, and one does not necessarily imply the other. A pCR is based on pathological findings after resection, commonly using the Dworak or Mandard tumour regression grading systems. A cCR is defined according to a combination of clinical examination (including digital rectal examination), radiological (in particular diffusion‐weighted MRI) and endoscopic appearances.

Following the initial description by Habr‐Gama and colleagues^2^, there are now a growing number of series reporting the use of neoadjuvant CRT as the sole treatment for rectal cancer that undergoes a cCR, resulting in further interest in the role of organ preservation in rectal cancer^3^. It is, however, important to be able to differentiate which tumours are more susceptible to undergoing a cCR. At present, the most reliable predictor of an increased response is tumour stage, with early tumours more likely to display a cCR. The use of CRT in combination with local excision is perhaps becoming better defined in early T1 rectal cancers, but its value in more advanced cancer is less clear^4^. The STAR‐TReC trial (ISRCTN14240288)^5^ will compare three different strategies for more advanced tumours up to T3b N0, and assess the feasibility of randomizing to a trial with organ preservation arms. However, the role of neoadjuvant CRT as sole treatment for even more locally advanced tumours that perhaps threaten the circumferential resection margin (CRM) is unknown, and it is likely that studies examining such tumours will need to incorporate the development of intensified CRT regimens.

Patients who have an apparent cCR may be offered entry into a watch‐and‐wait surveillance policy after a full and complete discussion. If patients are fit for intervention, salvage surgery is recommended for those who display tumour regrowth, which is most often luminal rather than nodal^1^. There is clearly an interest in both predicting patients who may undergo a cCR or pCR and/or improving cCR and/or pCR rates as there are currently no reliable clinical (apart from earlier stage), biochemical or molecular predictive biomarkers in clinical practice.

Radiotherapy (RT) is typically delivered via either a short‐ or long‐course strategy, the latter being employed to downstage tumours. A recent short study by the UK National Bowel Cancer Audit^6^ revealed that the median time from completion of CRT to surgical resection is currently 11 weeks in the UK, suggesting that the concept of delayed resection is gaining traction in clinical practice. A recent study^7^ suggested that increasing the interval between the end of CRT and surgical resection improves the response rate. Similarly, short‐course RT may be combined with a delayed interval to surgery; the recent Stockholm III trial^8^ has demonstrated improved tumour regression over traditional short‐course treatment.

**Figure 1:**
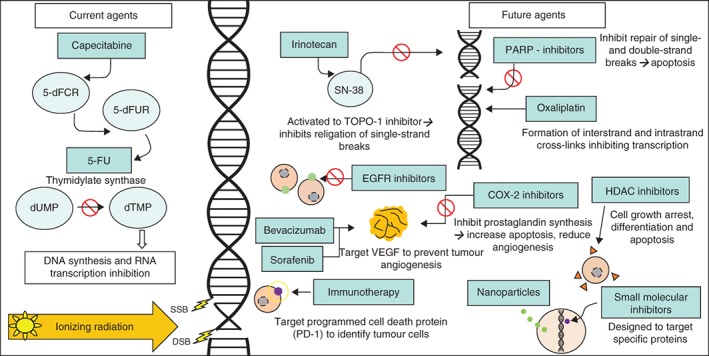
Summary of current and potential radiosensitizing agents. 5‐dFCR, 5′‐deoxy‐5‐fluorocytidine; 5‐dFUR, 5′‐deoxy‐5‐fluorouridine; 5‐FU, 5‐fluorouracil; dUMP, deoxyuridine monophosphate; dTMP, deoxythymidine monophosphate; SSB, single‐strand break; DSB, double‐strand break; TOPO, topoisomerase; EGFR, epidermal growth factor receptor; VEGF, vascular endothelial growth factor; PARP, poly(ADP‐ribose)polymerase; COX, cyclo‐oxygenase; HDAC, histone deacetylase

Radiosensitizers are employed routinely to improve the radiosensitivity of rectal cancer to RT; the standard of care is a concurrent single‐agent fluoropyrimidine. A number of studies have analysed novel agents or combination therapies that aim to improve radiosensitivity and cCR and/or pCR rates. The critical target for RT is DNA and the accumulation of DNA damage, particularly DNA double‐strand breaks, and the ability of tumour cells to repair this damage, contributes significantly to the therapeutic effect. Some agents and combination therapies (such as oxaliplatin, irinotecan and poly(ADP‐ribose) polymerase (PARP) inhibitors) might typically take advantage of this by creating additional DNA damage or inhibiting DNA damage repair, exacerbating the effects of RT. The aim of this review was to summarize the current and novel agents that have been employed in the treatment of LARC, and to consider their role in the context of cCR and organ preservation. A summary of radiosensitizing agents is provided in *Fig*. 1.

## Methods

**Figure 2:**
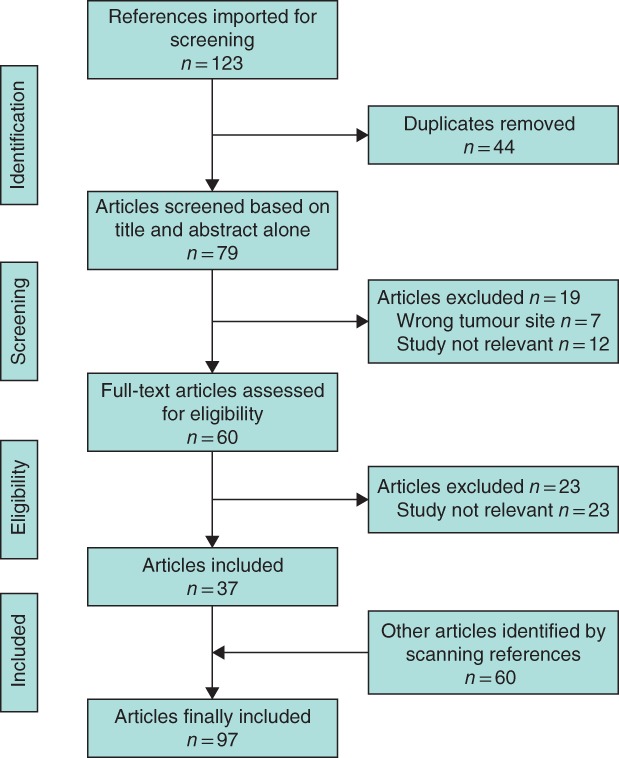
PRISMA diagram showing selection of articles for review

A literature search was performed for published full‐text articles using PubMed, Cochrane and Scopus databases using the search criteria string (‘radiosensitiser’ OR ‘radiosensitizer’) AND (‘rectal’ OR ‘rectum’) AND ‘cancer’ in November 2017. Additional papers were detected by scanning the references of relevant papers. Search results were initially included based on a relevant title, and these papers were then read in full. Inclusion criteria were: papers published in the English language, those with a focus on rectal cancer, all study types, and articles published between 1990 and 2017. Studies focusing on a primary malignancy other than rectal cancer were excluded. Two reviewers were involved at each stage, with search results being loaded into the Covidence system to enable joint reviews to take place methodically. Once eligible articles had been identified, a search was undertaken to exclude duplicated results or duplicated data sets to produce the final list of papers included (*Fig*. 2).

## Standard chemotherapy regimens

### 5‐Fluorouracil

5‐Fluorouracil (5‐FU) is an antimetabolite fluoropyrimidine. It is one of the most common chemotherapeutic agents used in cancer treatment, in particular breast and colorectal cancer. It was the first agent to be used as a radiosensitizer in conjunction with RT, predominantly in colorectal cancer. It works by inhibiting essential biosynthetic processes, and also by affecting cellular DNA and RNA functions. The mechanism of cytotoxicity of 5‐FU has been ascribed to the misincorporation of fluoronucleotides into RNA and DNA, and to inhibition of the nucleotide synthetic enzyme thymidylate synthase^9^.

There are a number of mechanisms by which 5‐FU could increase radiation sensitivity at the cellular level. One is thought to involve the killing of S‐phase cells, which are relatively radioresistant^10^, ^11^. This does not account for all of the increased radiation sensitivity produced by the drug because non‐cytotoxic concentrations can also increase sensitivity. Radiosensitization under non‐cytotoxic conditions occurs only when cells are incubated with the drug before and during radiation treatment. Several studies have suggested that 5‐FU should be given continuously during a course of fractionated radiation to achieve radiosensitization of most fractions^12^, ^13^. UK National Institute for Health and Care Excellence guidance^14^ focusing on stage III tumours, examining randomized comparisons of bolus *versus* infusional regimens, suggests that infusional therapy is equivalent to bolus treatment in terms of effectiveness, but has relatively reduced toxicity. Owing to concerns regarding the increased cost of infusional treatment and patient inconvenience, there remains geographical variation across the UK in the technique employed^14^, although 5‐FU has largely been superseded by oral capecitabine. Trials of 5‐FU are summarized in *Table* 1.

**Table 1:** Summary of fluoropyrimidine agents

						Results (%)*			
Reference	Phase	Disease stage	Test drug	Single/combination regimen	Cohort size	pCR rate	cCR rate	Other endpoints	Toxicity
15	III	T3–4 N–/+	5‐FU	Adjuvant RT *versus* RT + 5‐FU	204	n.a.	–	5‐year recurrence 41·5 (62·7)	Increased risk of GI or haematological problems with 5‐FU use; only 1 severe
16	III	T3–4 N+/–	5‐FU	Neoadjuvant RT *versus* RT + 5‐FU	773	11·4 (3·6)	–	5‐year LR 8·1 (16·5) 5‐year OS 67 (67)	Grade 3–4 toxicity 14·6 (3·6)
17	III	T3–4 N–/+	5‐FU	Neoadjuvant *versus* adjuvant RT + 5‐FU	267	15·0	0·8	5‐year DFS 64·7 (53·4) 5‐year OS 74·5 (65·6)	Grade 4 GI disturbance 24 (13)
18	III	T3–4 N–/+	5‐FU	Neoadjuvant RT *versus* RT + 5‐FU	1011	13·7 (5·3)	–	–	–
19	III	T3–4 N–/+	5‐FU	Neoadjuvant *versus* adjuvant RT + 5‐FU	823	8	–	5‐year LRR 6 (13) 5‐year DFS 68 (65) 5‐year OS 76 (74)	Grade 3–4 toxicity 27 *versus* 40
20	II	T3–4 N–/+	Capecitabine	Single	95	12	–	Downstaging 76	Grade 3 toxicity 3
21	II	T3–4 N–/+	Capecitabine	Single	54	18	–	Downstaging 51 Sphincter salvage 67	Grade 3–4 GI toxicity 2
22	II	T3–4 N–/+	Capecitabine	Single	31	7	–	Downstaging 54 3‐year DFS 60 3‐year OS 77	Grade 3–4 GI toxicity 36 Proctitis 32
23	III	T3–4 N–/+	Capecitabine	Capecitabine + oxaliplatin *versus* 5‐FU	42	24 (14)	–	Downstaging 81 (67)	Grade 3 GI toxicity 19 (14) Haematological 19 (14)
24	III	T3–4 N–/+	Capecitabine	Capecitabine *versus* 5‐FU	392	14 (5)	–	LR 6 (7) 5‐year OS 76 (67) 3‐year DFS 75 (67)	Grade 3–4 GI toxicity 9 (2)
25	III	T3–4 N–/+	Capecitabine	Capecitabine +/– oxaliplatin *versus* 5‐FU +/– oxaliplatin	1608	20·7 (17·8)	–	Downstaging 21·1 (21·3) Sphincter salvage 59·3 (59·4)	Grade 3–5 GI toxicity 11·7 (11·7) Addition of oxaliplatin increased GI disturbance (*P* < 0·001)

* Results for control group are shown in parentheses. pCR, pathological complete response; cCR, clinical complete response; 5‐FU, 5‐fluorouracil; RT, radiotherapy; n.a., not applicable; GI, gastrointestinal; LR, local recurrence; OS, overall survival; DFS, disease‐free survival; LRR, locoregional recurrence.

Krook and colleagues^15^ first conducted an RCT to assess adjuvant RT with or without systemic bolus 5‐FU chemotherapy, and confirmed an improvement in local relapse rates with a survival benefit in favour of 5‐FU‐based RT in comparison with RT alone. The phase III French Federation Francophone de Cancerologie Digestive (FFCD) 9203 study^16^ randomized patients with stage II–III rectal cancer to receive RT alone or with infusional 5‐FU/leucovorin. Patients in both arms subsequently underwent surgery and four cycles of 5‐FU/leucovorin. The preoperative chemoradiation arm showed a significant improvement in pCR rate (11·4 *versus* 3·6 per cent; *P* < 0·050) and local relapse rate (8·1 *versus* 16·5 per cent; *P* < 0·050). The 5‐year survival in both arms was 67 per cent.

The National Surgical Adjuvant Breast and Bowel Project (NSABP) R‐03 phase III study^17^ compared the use of neoadjuvant and adjuvant CRT in patients with T3–T4 or node‐positive rectal cancer using 5‐FU and leucovorin. Those receiving neoadjuvant therapy had a pCR rate of 15·0 per cent and 5‐year disease‐free survival (DFS) rate of 64·7 per cent. Among those undergoing adjuvant CRT, 39·2 per cent had sphincter‐saving surgery (*versus* 47·8 per cent in the neoadjuvant cohort) and a DFS rate of 53·4 per cent. Five‐year overall survival rates were 74·5 and 65·6 per cent in the neoadjuvant and adjuvant treatment groups respectively, supporting the use of CRT before rather than after operation. In a 2012 Cochrane review, Petersen and colleagues^26^ considered the use of 5‐FU for additional adjuvant chemotherapy. The pooled data from 21 RCTs, including almost 10 000 patients, found improved DFS and overall survival with use of adjuvant chemotherapy. Owing to lack of tumour stage‐specific data, however, it was not possible to draw a link of benefit to specific locally advanced tumours, potentially indicating an area for further work.

The phase III European Organisation for Research and Treatment of Cancer (EORTC) 22921 study^18^ randomized patients with stage II–III rectal cancer to neoadjuvant RT alone *versus* RT with concurrent bolus 5‐FU/leucovorin, with subsequent randomization to postoperative chemotherapy or not. The authors concluded that adding 5‐FU‐based chemotherapy, either before (as part of CRT) or after operation, conferred a significant advantage in terms of local control.

The German Rectal Cancer Study Group^19^ randomly assigned 823 patients with clinical stage T3 or T4 or node‐positive disease to receive either preoperative or postoperative CRT. The results showed a significantly lower 5‐year cumulative incidence of local relapse in favour of the preoperative treatment group (6 *versus* 13 per cent; *P* = 0·006). Five‐year DFS (68 *versus* 65 per cent) and overall survival (76 *versus* 74 per cent) rates were similar. Significant tumour downstaging was seen after preoperative combined treatment, with a pCR rate of 8 per cent.

In a pooled analysis of 5‐FU phase II–III trials^27^ including 3157 patients, the pCR rate was 13·5 per cent. On multivariable analysis, statistically significant factors for a higher pCR rate were the addition of a second chemotherapy agent and the method of continuous infusion.

### Capecitabine

The development of an oral 5‐FU drug was driven by the desire to overcome the perceived limitations associated with intravenous infusion of 5‐FU, such as extended hospital stay, the need for intravenous lines and associated healthcare costs. Capecitabine (Xeloda®; Roche, Basle, Switzerland) is an oral prodrug of 5‐FU; it is a fluoropyrimidine carbamate that undergoes a three‐step *in vivo* enzymatic conversion to 5‐FU. The final step is mediated by the enzyme thymidine phosphorylase, which is upregulated in tumour tissue compared with adjacent healthy tissue. This theoretically allows selective activation of the drug and low systemic toxicity^28^. After oral administration, capecitabine passes rapidly and extensively through the intestinal membrane as an intact molecule. Capecitabine is not cytotoxic itself; the only cytotoxic moiety is 5‐FU, which is generated preferentially in human cancer cells. Preferential activation of capecitabine to 5‐FU in malignant tumour was demonstrated in animal models bearing human xenografts^29^. *Table* 1 provides an overview of clinical trials of capecitabine.

The first phase II trials^20^, ^21^ showed that RT plus capecitabine is well tolerated and easier to administer than protracted intravenous infusion of 5‐FU, with a pCR rate comparable to intravenous infusion of 5‐FU for LARC. In 2008, another phase II trial^22^ of 31 patients with LARC showed that capecitabine was well tolerated orally and had radiosensitizing effects comparable to those of neoadjuvant 5‐FU therapy. In 2011, Swellengrebel *et al*.^30^ again showed that oral capecitabine had an acceptable acute toxicity profile in a cohort of 147 patients with LARC. In 2015, Saha and co‐workers^23^ conducted a randomized control pilot study comparing capecitabine–oxaliplatin (CAPOX) as a radiosensitizer with 5‐FU–leucovorin; the two arms were comparable in terms of objective response rate, pCR rate, R0 resection and toxicity profile^23^. Noh and colleagues^31^, ^32^ investigated different timings for administration of capecitabine among 171 patients undergoing RT followed by total mesorectal excision (TME) 4–6 weeks after neoadjuvant therapy and assessed the radiosensitization response by pCR. The optimal radiosensitizing effects of capecitabine were achieved if it was administered 1 h before RT.

A phase III RCT^24^ between 2002 and 2007 recruited nearly 400 patients with stage II and III LARC, comparing CRT with capecitabine *versus* 5‐FU. The primary endpoint was 5‐year overall survival, which in the capecitabine group was non‐inferior to that in the 5‐FU group (76 *versus* 67 per cent; *P* < 0·001). The local recurrence rate was similar in the two groups (6 *versus* 7 per cent); however, the rate of distant metastasis was 9 per cent lower in the capecitabine group, with increased 3‐year DFS.

The phase III NSABP R‐04 trial^25^ aimed to ascertain the optimal neoadjuvant chemotherapy regimen alongside RT for stage II–III rectal cancer. Infusion of 5‐FU and oral capecitabine with or without intravenous oxaliplatin were compared in 1608 patients. The pCR rate was 17·8 per cent for those receiving 5‐FU and 20·7 per cent among those receiving capecitabine. Sphincter salvage rates were largely comparable between the groups at 59·4 and 59·3 per cent respectively, as were rates of tumour downstaging (21·3 *versus* 21·1 per cent). The addition of oxaliplatin led to a small increase in pCR rate, but a reduction in sphincter salvage and downstaging, and a significant increase in toxicity.

## Additional chemotherapy agents to enhance radiosensitivity

### Oxaliplatin

Oxaliplatin is a third‐generation platinum‐based drug that enhances radiation‐induced cytotoxicity via irreparable DNA damage through formation of interstrand and intrastrand crosslinks, induction of G2/M cell‐cycle arrest, blockage of DNA replication and inhibition of transcription^33^, ^34^. Preclinical data indicated potent radiosensitizing properties of the drug, with synergism between oxaliplatin and RT^35^, ^36^; these findings have been applied to several clinical trials for patients with LARC.

Phase I–II studies focusing on the addition of oxaliplatin to 5‐FU‐based neoadjuvant CRT reported promising results^33^. pCR rates varied between 7 and 28 per cent, compared with 8–15 per cent in the 5‐FU‐alone group^34^, ^37^. Single weekly dosing was the most effective regimen, with diarrhoea and neuropathy the most commonly reported adverse effects.

Six large phase III trials to date have compared fluoropyrimidine CRT with or without oxaliplatin. The results from these trials are summarized in *Table* 2. The ACCORD 12/prodige 2 trial^38^ randomized 598 patients to standard capecitabine‐based neoadjuvant CRT or additional weekly dosing with oxaliplatin together with an increased radiation dose. The difference in pCR rate of 13·9 *versus* 19·2 per cent was not significant (*P* = 0·09), although ACCORD had been powered to detect an increase from 11 to 20 per cent with CAPOX. There was an increase in grade 3–4 toxicity with oxaliplatin.

**Table 2:** Summary of other chemotherapy agents

						Results (%)*			
Reference	Phase	Disease stage	Test drug	Single/combination	Cohort size	pCR rate	cCR rate	Other endpoints	Toxicity
38	III	T3–4 N–/+	Oxaliplatin	Capecitabine + oxaliplatin *versus* capecitabine alone	598	19·2 (13·9)	0·7 (0)	Positive CRM 9·9 (19·3)	Grade 3–4 toxicity 25 (1)
39	III	T3–4 N–/+	Oxaliplatin	5‐FU + oxaliplatin *versus* 5‐FU alone	1236	17 (13)	–	3‐year DFS 75·9 (71·2)	Grade 3–4 toxicity 23 (20)
40	III	T3–4 N–/+	Oxaliplatin	5‐FU +/– oxaliplatin *versus* capecitabine +/– oxaliplatin	1608	–	–	LR 11·2 (11·8) 5‐year DFS 66·4 (67·7) 5‐year OS 81·3 (79)	Addition of oxaliplatin significantly increased toxicity (*P* < 0·001)
41	III	T3–4 N–/+	Oxaliplatin	Oxaliplatin +5‐FU *versus* 5‐FU alone	1094	–	–	3‐year DFS 74·5 (73·9)	‐
42	III	T3–4 N–/+	Oxaliplatin	5‐FU + oxaliplatin *versus* 5‐FU alone	747	16 (16)	–	Positive CRM 7 (4)	Grade 3–4 toxicity 24 (8) Discontinued owing to toxicity 17 (4)
43	III	T3–4 N–/+	Oxaliplatin	5‐FU + oxaliplatin *versus* 5‐FU alone	495	27·5 (14·0)	–	Negative nodes 87·4 (80·1)	Grade 3–4 haematological toxicity 19 (12·9)
44	I–II	T3–4 N–/+	Irinotecan	Irinotecan +5‐FU	59	25	–	Downstaging 41 3‐year DFS 40	Grade 3–4 toxicity 28·8
45	II	T3–4 N–/+	Irinotecan	Irinotecan + capecitabine	36	15	–	3‐year OS 80	Grade 3–4 haematological toxicity 25
46	II	T3–4 N–/+	Irinotecan	Irinotecan + capecitabine	48	25	–	5‐year DFS 75 5‐year OS 94	Grade 3 toxicity 10·5 No grade 4 toxicity
47	II	T3–4 N–/+	Irinotecan	Irinotecan + 5‐FU *versus* 5‐FU alone	106	26 (30)		Downstaging 75 (74) 5‐year OS 75 (61) 5‐year DFS 85 (78)	Grade 3 toxicity 13 (8)
48	II	T3–4 N–/+	Irinotecan	Irinotecan + capecitabine	110	21·8	–	Negative CRM 89·1 3‐year DFS 96·9 3‐year OS 88·2	Grade 3 GI toxicity 22 No grade 4 toxicity

* Results for control group are shown in parentheses. pCR, pathological complete response; cCR, clinical complete response; CRM, circumferential resection margin; 5‐FU, 5‐fluorouracil; DFS, disease‐free survival; LR, local recurrence; OS, overall survival; GI, gastrointestinal.

The CAO/ARO/AIO‐04 study^39^, which included over 1000 participants, was the only trial to find a significantly improved pCR rate with oxaliplatin (from 13 to 17 per cent; *P* = 0·038). This was also the only trial to report an advantage for oxaliplatin in terms of 3‐year DFS (71·2 to 75·9 per cent). There were no significant differences in grade 3–4 toxicities or postoperative complications. However, the infusional 5‐FU regimen was changed between the control and experimental arms, with oxaliplatin being added to 16 weeks of postoperative adjuvant chemotherapy compared with 16 weeks of bolus 5‐FU alone in the control arm; this means that the relative contributions of oxaliplatin in CRT compared with adjuvant chemotherapy are difficult to define.

The NSABP R‐04^40^ and PETACC‐6^41^ trials, published only in abstract form, found that addition of oxaliplatin to 5‐FU‐based neoadjuvant therapy led to decreased treatment compliance and increased toxicity, with no associated improvement in pathological tumour downstaging. The STAR‐01 trial^42^ randomized 747 patients to standard 5‐FU chemotherapy or additional oxaliplatin. Interim analysis detected no difference in pCR and toxicity problems with the addition of oxaliplatin^42^.

Interestingly, initial results from the Chinese FOWARC trial^43^ showed that use of a modified FOLFOX (oxaliplatin, leucovorin, 5‐FU) 6 regimen in addition to 5‐FU + RT gave significantly improved rates of pCR compared with single‐agent 5‐FU + RT (27·5 *versus* 14·0 per cent respectively). The trial also showed comparable downstaging and acceptable toxicity in patients with stage II–III disease, and good compliance. Long‐term data are awaited and may still be important for future practice.

The evidence at present, including subsequent meta‐analyses^49^, ^50^, still supports the use of a single‐agent fluoropyrimidine as the standard of care because of a lack of consistent improvement in pCR and 3‐year DFS rates^51^ with the combined regimen, and the greater toxicity due to oxaliplatin^52^.

### Irinotecan

Irinotecan, a topoisomerase (TOPO) 1 inhibitor, inhibits religation of single‐strand DNA breaks through the formation of camptothecin 11–TOPO‐1–DNA complexes^53^. A preclinical study^54^ has demonstrated irinotecan to be not only a feasible addition to 5‐FU chemotherapy, but also a potent radiosensitizing agent in colorectal cancer, even under hypoxic conditions.

In a small phase I trial in 2008, Choi *et al*.^55^ examined the addition of weekly irinotecan to traditional 5‐FU neoadjuvant CRT in 16 patients with locally advanced T3–T4 rectal cancers. Some 94 per cent of patients were eligible to progress with surgical resection at the time of restaging, with 93 per cent achieving a R0 resection. In eight patients the disease was downstaged based on the TMN classification, with a pCR rate of 25 per cent. Although the numbers were too small to draw any firm conclusions, the evidence was promising in terms of combination potential.

Mehta and colleagues^56^ conducted a phase II trial with the same dosing strategy in a cohort of 32 patients, of whom 38 per cent achieved a pCR and 71 per cent TNM downstaging. However, 56 per cent experienced acute toxicity with an initial dose of 50 mg/m^2^, requiring dose alteration or delay in administration. Overall, small phase II studies^44^, ^45^, ^46^, ^56^ focusing on this approach have achieved pCR rates of 14–37 per cent and tumour downstaging in 24–71 per cent. A summary of these trials is provided in *Table* 2.

Mohiuddin *et al*.^47^ reported 5‐year outcomes on 106 patients randomized to either basic 5‐FU CRT or additional 4‐week doses of 50 mg/m^2^ irinotecan. They reported an increase in overall survival of 14 per cent with the addition of irinotecan; however, the locoregional recurrence rate was 17 per cent, compared with 16 per cent without irinotecan, and respective distal recurrence rates were 21 and 16 per cent. There was no significant difference between the treatment arms in terms of pCR or downstaging, but an increased rate of acute toxicity was reported in the irinotecan group. Gollins and colleagues^48^ reported on 110 patients with MRI‐defined locally advanced rectal cancer threatening or involving the surgical CRM treated with a regimen of irinotecan 60 mg/m^2^ weekly for the first 4 weeks of a 5‐week course of capecitabine CRT. In total, 24 patients (21·8 per cent) had a pCR and 98 (89·1 per cent) a negative CRM. A further study focusing on long‐term outcome in 115 patients^57^ found no significant difference between the two treatment arms in terms of pCR, with a higher overall survival rate of 87 per cent and DFS rate of 79 per cent in the irinotecan group (median follow‐up 60 months).

Despite the promise of the above studies, no phase III trial of concurrent irinotecan has yet been reported^58^. This will be rectified in the future by the ongoing UK ARISTOTLE trial, which will complete accrual (target 600 patients) in mid‐2018. In MRI‐defined high‐risk rectal cancer, ARISTOTLE will compare CRT with concurrent capecitabine with or without irinotecan.

## Epidermal growth factor receptor inhibitors

Epidermal growth factor receptor (EGFR), a member of the ErbB family of receptors, is relevant in colorectal cancer because overexpression or upregulation of the *EGFR* gene occurs in 60–80 per cent of cases^59^, ^60^, ^61^. Expression of the gene is also associated with poor survival^62^, ^63^, ^64^. The anti‐EGFR monoclonal antibodies cetuximab and panitumumab are already approved for the treatment of RAS wild‐type metastatic colorectal cancer^65^, but their role in LARC remains unclear.

There have been several clinical trials of EGFR‐targeting monoclonal antibodies as radiosensitizers in neoadjuvant therapy for LARC. These trials are summarized in *Table* 3. Early efficacy results in terms of pCR rate were around 5–10 per cent^74^, ^75^, ^76^. However, these studies did not investigate tumour RAS status, which is used as a predictive biomarker for anti‐EGFR monoclonal antibody response in metastatic colorectal cancer^77^, ^78^. Potentially, optimal ordering of chemotherapy, RT and the EGFR inhibitor might unlock the full radiosensitizing potential of anti‐EGFR monoclonal antibodies^79^.

**Table 3:** Summary of phase II trials of epidermal growth factor receptor inhibitors

						Results (%)*			
Reference	Phase	Disease stage	Test drug	Single/combination	Cohort size	pCR rate	cCR rate	Other endpoints	Toxicity
66	II	T3–4 N–/+	Panitumumab	Single	19	0	–	Downstaging 41 Negative CRM 76 LRC 90 DFS 79	GI disturbance 89 Grade 4 toxicity 21
67	II	T3–4 N–/+	Cetuximab	Cetuximab + capecitabine	31	0	–	Downstaging 42	GI disturbance 13 Grade 4 toxicity 3
68	II	T3–4 N–/+	Panitumumab	Panitumumab + oxaliplatin +5‐FU	60	21	–	Downstaging 58	GI disturbance 39 1 death
69	II	T3–4 N–/+	Panitumumab	Panitumumab + capecitabine *versus* capecitabine alone	68	10 (18)	–	R0 resection 85 (93) Sphincter salvage 69 (70) Downstaging 87 (85)	GI disturbance 10 (4)
70	II	T3–4	Cetuximab	Cetuximab + capecitabine + oxaliplatin *versus* capecitabine + oxaliplatin	165	11 (9)	11 (7)	Radiological response 71 (51)	GI disturbance 8 (9)
71	II	T2–4 N–/+	Cetuximab	Cetuximab + capecitabine + irinotecin	82	17	5	R0 resection 82	GI disturbance 25 Grade 4 toxicity 10
72	II	T3–4 N–/+	Cetuximab	Cetuximab + capecitabine	47	8	–	3‐year DFS 72 3‐year RFS 74 3‐year OS 68	2 of 32 unable to complete treatment owing to GI disturbance and leucopenia
73	I–II	T3–4 N–/+	Cetuximab	Cetuximab + capecitabine + oxaliplatin	60	8	–	5‐year OS 76 3‐year DFS 88 5 ‐year CSS 78	Grade 2 toxicity 5

* Results for control group are shown in parentheses. pCR, pathological complete response; cCR, clinical complete response; CRM, circumferential resection margin; LRC, locoregional control; DFS, disease‐free survival; GI, gastrointestinal; 5‐FU, 5‐fluorouracil; RFS, relapse‐free survival; OS, overall survival; CSS, cancer‐specific survival.

A phase II trial^66^ was designed to assess the pCR (primary endpoint) following neoadjuvant therapy with panitumumab and RT. Of 19 enrolled patients, 17 were evaluable for pathology assessment. Although no pCR was observed, seven patients (41 per cent) had grade 3 Dworak pathological tumour regression. As the primary endpoint was not achieved, the authors were unable to make any recommendation for the use of panitumumab in treatment of LARC. Similar findings were reported in other phase II trials where the primary endpoint of pCR was not achieved^67^, ^68^, and toxicity was high^69^.

EXPERT‐C^70^ was a randomized phase II trial of neoadjuvant CAPOX with or without cetuximab, followed by capecitabine‐based CRT with or without cetuximab, followed by surgery and then adjuvant CAPOX with or without cetuximab in 165 high‐risk patients with rectal cancer. Cetuximab did not improve the primary outcome (pCR), so it was not felt to have contributed significantly to increased radiation‐induced cytotoxicity. The EXPERT‐C trial did, however, find that TP53 tumour suppressor protein wild‐type status was a predictive biomarker in favour of cetuximab‐based therapy.

The prospective phase II EXCITE trial^71^, published in 2017, focused on the addition of cetuximab to an irinotecan–capecitabine‐based neoadjuvant CRT regimen in 82 patients. Fourteen patients (17 per cent) had a pCR. As a side point of interest, contrary to the planned protocol, four patients achieved an endoscopically and MRI‐confirmed cCR, and were managed using the emerging watch‐and‐wait approach. Overall 24 patients (29 per cent) had an excellent clinical or pathological response. Using next‐generation sequencing, 46 per cent of matched biopsy–resection specimens were discrepant for EGFR pathway mutations. Intratumoral heterogeneity was suggested as a possible explanation, manifesting as a geographical biopsy miss or chemoradiation‐driven emergence of new mutations.

Phase II studies so far have failed to suggest a benefit in terms of pCR rate and DFS, and have shown no consistent correlation with KRAS status^72^, ^73^, ^80^. There is currently no role for the addition of EGFR‐targeted therapy as a radiosensitizer in the treatment of LARC^81^. However, a pilot study^82^ of RT with personalized chemotherapy and biological therapy, based on molecular markers among 16 patients with T3 or N1 rectal cancers, showed a pCR rate of 50 per cent, which may be the basis for future molecular guided studies.

## Antiangiogenesis therapy

### Bevacizumab

Bevacizumab is a monoclonal antibody that targets vascular epithelial growth factor (VEGF). In combination with cytotoxic chemotherapy, it has shown potential for rectal cancer treatment; the evidence is, however, currently limited to phase I–II trials^83^. Salazar *et al*.^84^ undertook a multicentre randomized phase II trial in 90 patients with LARC, of capecitabine with or without bevacizumab. The pCR rate was 16 per cent in the bevacizumab arm, compared with 11 per cent in the control arm, and an additional 20 per cent of tumours were downstaged. However, the predefined efficacy endpoint of a difference in treatment arms of 10 per cent was not met, despite these encouraging results.

Landry *et al*.^85^ performed a phase II trial of the addition of bevacizumab therapy with 5‐year follow‐up. Of 57 patients included in the data analysis, 17 per cent achieved a pCR, with an overall 5‐year survival rate of 80 per cent and relapse‐free survival rate of 81 per cent. The pCR endpoint of 30 per cent was not reached and, owing to substantial side‐effects (1 death was attributed to study therapy), the regimen was not considered worthy of further work by the authors.

### Sorafenib

Sorafenib is a multikinase inhibitor that blocks the receptor tyrosine kinase of VEGF, platelet‐derived growth factor and the RAF serine–threonine kinases along the RAF–mitogen‐activated protein kinase kinase–extracellular signal‐related kinase pathway. Jeong and colleagues^86^ assessed its potential as a radiosensitizer using three colorectal cell lines, and a xenograft animal model. They were able to demonstrate a scientific rationale for combination therapy, with enhanced radiosensitivity being shown in all three cell lines and the xenograft model, and delayed DNA damage repair caused by the radiation treatment. Van Moos *et al*.^87^ evaluated its effect in a cohort of 54 patients with KRAS‐mutated rectal tumours in combination with capecitabine‐based CRT. The pCR rate was 60 per cent, with downstaging in 82 per cent. A second phase I study^88^ also produced encouraging results, with a pCR of 36 per cent.

## Poly(ADP‐ribose) polymerase inhibition

PARPs, particularly PARP‐1, play a critical role in the recognition and repair of DNA single‐ and double‐strand breaks. Higher PARP activity has been noted in cancer cells with increased proliferation and chemoradioresistance, and this has led to the development of PARP inhibitors, which reduce the cancer cell's ability to repair single‐ and double‐strand breaks generated by RT and lead to cell death. Preclinical trials have demonstrated radiosensitizing effects in multiple colorectal cell lines^89^, although this effect is potentially largely dependent on the status of oncogenes such as *BRCA1*/*BRCA2* 90 generating defective DNA double‐strand break repair (termed synthetic lethality), and dysregulation of P53^91^.

Veliparib (ABT‐888), a potent orally bioavailable PARP‐1/2 inhibitor, has been shown to enhance the antitumor activity of chemotherapy and RT in preclinical colorectal cancer models^92^. In *in vitro* and *in vivo* experiments in colorectal cancer, veliparib had independent radiosensitization effects and was synergistic with chemotherapy, especially with irinotecan. Final results from a phase Ib dose‐escalation study of veliparib plus capecitabine‐based CRT and surgery were published in 2017^93^, demonstrating a pCR rate of 28 per cent. As with the EGFR monoclonal antibodies, this class of potential radiosensitizer remains an area of interest and future studies are needed to elucidate its role in rectal cancer. Potential predictive biomarkers have not been identified. A recent report^94^ has described pharmacodynamic assays that are able to measure the low levels at which PARP inhibitors are active.

## Immunotherapy

The immune system plays an intricate and complex role in all aspects of cancer from carcinogenesis to treatment^95^. Over the past 10 years, a great deal of work has been done to better understand that role, with the development of therapies such as programmed cell death protein 1 (PD‐1)/programmed death ligand 1 (PD‐L1) inhibitors, cancer vaccines and adoptive cell therapy. In a phase I–II trial^96^ of adjuvant immunotherapy involving sentinel lymph node T lymphocytes in 55 patients with metastatic colorectal cancer, there was no treatment‐related toxicity, and 24‐month survival rates were 56 and 18 per cent in the treatment and control groups respectively.

Use of cytokine therapy, although very early in terms of research into colorectal cancer, has been approved by the US Food and Drug Administration for melanoma and renal cell carcinoma (interleukin 2). Although much of the work focusing on colorectal malignancy is in its early phases (I–II), there is evidence to suggest the potential use of these therapies in a combination role for a correctly selected cohort^97^. Specifically, the PD‐1 immune checkpoint inhibitors pemrolizumab and nivolumab have shown promising activity in DNA mismatch repair‐deficient (dMMR)/microsatellite instability – high colorectal cancers, which carry a high mutation load and an active immune microenvironment^98^, ^99^. A small proportion of rectal cancers are dMMR, but one possible area of research is to determine whether the proinflammatory properties of RT might enhance the response of microsatellite‐stable tumours to PD‐1 blockade. The R‐IMMUNE phase II study^100^ is currently recruiting to compare the use of atezolizumab as a radiosensitizer with 5‐FU‐based neoadjuvant CRT. More studies are in the pipeline, with a recent UK proposal aiming to assess the effect of the PD‐L1 inhibitor durvalumab in combination with RT.

## Novel agents

With a clear focus of research on optimizing neoadjuvant therapy, several novel agents ranging from cyclo‐oxygenase 2 inhibitors to nanoparticles have been investigated in the preclinical setting (*Table* 4). Further phase I studies are in preparation to examine both prostaglandin E2 receptor inhibitors (PRAER 1 trial) and Ad3/Ad11p chimeric adenoviruses (CEDAR trial).

**Table 4:** Summary of novel radiosensitizing agents

Reference	Study design	Findings
COX‐2 inhibitors	Cox‐2 is an inducible enzyme that regulates prostaglandin synthesis and is overexpressed at sites of inflammation and in epithelial malignancy tumours^101^. It is involved in the regulation of apoptosis, angiogenesis and tumour cell invasiveness. Preclinical studies suggest the potential of COX‐2 inhibitors as selective radiosensitizers^102^	
Debucquoy *et al*.^103^	Double‐blind randomized phase II; in addition to 5‐FU; 35patients	Improved downstaging No increased toxicity
Nanoparticles	Aim to improve the therapeutic index of chemoradiotherapy and overcome potential systemic excess toxicity. Focus on particle size sub‐50 nm	
Caster *et al*.^104^	Particles 50, 100 and 150 nm in size loaded with 2 DNA repair inhibitor model drugs in colorectal cancer cell lines	All sizes potent radiosensitizers Good toxicity tolerance
Tian *et al*.^105^	CRLX101 in combination with oxaliplatin and 5‐FU	Increased efficacy of chemoradiotherapy Early stage; needs expansion
Histone deacetylase inhibitors	Emerging therapeutic concept attempting to target epigenetic regulatory mechanisms and act as a radiosensitizer in combination therapy. SAHA approved as a single agent for refractory cutaneous T‐cell lymphoma	
Folkvord *et al*.^106^	Preclinical study of SAHA using 2 xenograft models	*In vitro*: improved radiosensitivity (*P ≤* 0·050) across cell lines at all radiation doses less than 6 h after exposure *In vivo*: pCR achieved in 1 model
Saelen *et al*.^107^	Vorinostat assessed under hypoxic conditions *in vitro*	Enhanced radiosensitivity across cell lines Warrants further research
Small molecular inhibitors	Low molecular weight; able to target both extracellular and intracellular proteins	
Kleiman *et al* 108	Preclinical Focus on radiosensitizers for KRAS mutant tumours 28 known radiosensitizers assessed	6 effective; AZD7762 most highly potent Suggested investigation into role of CHK2 inhibitors
Nelfinavir	HIV protease inhibitor; inhibits Akt at standard clinical doses and results in radiosensitivity	
Hill *et al*.^109^	Non‐randomized SONATINA clinical trial focusing on safety in 10 patients with T3–4 N0–2 M1 rectal cancers recruited over 2 years 14 days total oral treatment (7 days preoperative)	2 discontinued owing to toxicity 5 grade 3 toxicity Warrants further research
Buijsen *et al*.^110^	Phase I trial including 12 patients Escalating doses with capecitabine Primary endpoint: dose‐limiting toxicity	4 of 6 experienced toxicity, precluding further dose escalation pCR 27% Further toxicity concerns
Zerumbone	Cyclic sesquiterpene from rhizomes of edible ginger plant; emerging evidence of potential for inhibition of proliferation of human colonic adenocarcinoma cells, with minimal toxicity^111^	
Deorukhkar *et al*.^112^	3 colorectal cancer cell lines Inhibition of proliferation identified in dose‐dependentmanner	Marked radiosensitizer in clonogenic survival curves Little effect on normal fibroblasts Warrants further research
Bortezomib	Modified dipeptidyl boronic acid derived from leucine and phenylalanine that acts as a 26S proteasome inhibitor. The ubiquitin–proteasome pathway is involved in intracellular protein degradation in eukaryotic cells	
O'Neil *et al*.^113^	10 patients with stage II or III rectal cancer received 5‐FU‐based chemoradiotherapy plus bortezomib twiceper week	pCR 10% High toxicity – diarrhoea Study not progressed

COX, cyclo‐oxygenase; 5‐FU, 5‐fluorouracil; SAHA, suberoylanilide hydroxamic acid; pCR, pathological complete response; CHK2, serine–threonine kinase 2; HIV, human immunodeficiency virus.

## Alternatives to standard radiotherapy strategies

### Dose escalation

An alternative potential method of enhancing the effectiveness of CRT is by increasing the radiation dose, via an increased external‐beam dose or endocavitary brachytherapy. There is evidence to suggest that a dose–response relationship with pCR exists^114^.

A prospective single‐centre study^115^ from Denmark in patients with T2–3 cancers within 6 cm of the anal verge used radiation dose intensification to the primary tumour delivered with intensity‐modulated external‐beam RT to 60 Gy in 30 fractions over 6 weeks, with 50 Gy to the pelvic nodes, combined with an endorectal brachytherapy tumour boost to 5 Gy and tegafur/uracil on treatment days. Of the 51 patients treated, 78 per cent achieved a cCR and organ preservation; the local recurrence rate was 26 per cent at 2 years. Grade 3 diarrhoea occurred in 8 per cent, and long‐term rectal bleeding was of concern during follow‐up.

Gerard and colleagues^116^ demonstrated improved clinical (24 *versus* 2 per cent) and pathological (57 *versus* 34 per cent) responses using the 50‐Kv Papillon technique for contact X‐ray brachytherapy (CXB). Patients with a clinical incomplete response to external‐beam CRT have been shown to achieve a cCR after a CXB boost, with only 11 per cent developing recurrence^117^.

With both approaches, there is a lack of randomized data. The recently funded UK APHRODITE study will randomize patients with T1–T3b rectal adenocarcinomas with a maximum diameter of 4 cm, considered unsuitable for radical TME surgery, to standard CRT *versus* RT dose‐escalated CRT. The OPERA trial will randomize patients with early cT2–T3a–b tumours smaller than 5 cm in diameter, treated with external‐beam CRT, to either an external‐beam CRT boost or a CXB boost.

### Delivery modification

An alternative strategy to dose escalation is the development of novel delivery methods that reduce toxicity, particularly to the small bowel. Intensity‐modulated RT is one such technique that has been proposed owing to its highly conformal dose distribution. There are currently few published prospective data to support its routine use; however, a recent meta‐analysis^118^ of retrospective studies has suggested that it has a significantly lower toxicity profile than routine three‐dimensional CRT. Future developments may ultimately lead to traditional photon irradiation being replaced with charged particles such as protons or carbon ions, which may have even greater biological effectiveness while maintaining a favourable toxicity profile. At the present time, further clinical studies and access to treatment facilities are required to assess the applicability of these techniques fully^119^, ^120^.

### Preoperative chemotherapy given sequentially with (chemo)radiotherapy

The twofold rationale for giving neoadjuvant chemotherapy sequentially, either before or after (C)RT, followed by surgery, is to improve the response of the primary tumour and to reduce the distant metastasis rate. Owing to morbidity from RT and pelvic surgery, individuals who have undergone preoperative CRT then surgery may fail to start adjuvant chemotherapy or tolerate it poorly, resulting in dose reductions^121^. A meta‐analysis^122^ of four trials including preoperative RT, however, has questioned the benefit of postoperative chemotherapy (hazard ratio for DFS 0·91, 95 per cent c.i. 0·77 to 1·07; *P* = 0·230), possibly for this reason. Giving chemotherapy before surgery allows an increased dose intensity to be delivered, potentially increasing the response rate.

However, although the concept of ‘total neoadjuvant therapy’ is gaining traction^123^, there is currently very little randomized phase II (and no phase III) evidence specifically examining the benefit of neoadjuvant chemotherapy. Grupo Cancer de Recto (GCR) 3^124^ was a randomized phase II study of preoperative CAPOX followed by CRT then surgery *versus* CRT then surgery then postoperative CAPOX in 108 patients. Less toxicity (*P <* 0·001) and better compliance (*P* < 0·001) were demonstrated for the same regimen used as neoadjuvant chemotherapy compared with adjuvant chemotherapy, although the pCR rate was no different (13 *versus* 14 per cent respectively).

A non‐randomized US–Canadian trial^125^ examined four sequential study groups of patients with LARC, examining CRT followed by chemotherapy then surgery. Group 1 had CRT followed by TME 6–8 weeks later. Groups 2, 3 and 4 had two, four and six 2‐weekly cycles of modified FOLFOX delivered between CRT and TME. The pCR rate was 18, 25, 30 and 38 per cent for groups 1–4 respectively. Although promising, it is not clear whether the increased downstaging occurred because of a greater gap between CRT and surgery (6, 8, 12 and 16 weeks for groups 1–4 respectively).

Randomized studies are urgently needed to examine the efficacy of intensified neoadjuvant CRT regimens for rectal cancer, including the sequential addition of preoperative chemotherapy, in comparison to standard neoadjuvant CRT alone.

### Neoadjuvant chemotherapy alone

In the modern TME era, local recurrence rates have fallen to as low as 5 per cent. However, CRT has not affected distant metastatic relapse, which affects up to 30 per cent of patients. Although surgery is associated with long‐term sexual, bowel and bladder dysfunction, preoperative RT can exacerbate this morbidity^126^. Consideration should be given to whether chemotherapy alone can be as effective as CRT in terms of DFS, thereby avoiding some acute and long‐term toxicity.

Several small single‐arm studies using mainly oxaliplatin‐based chemotherapy have reported promising DFS rates. In addition, studies^127^, ^128^ examining neoadjuvant CAPOX followed by CRT have clearly shown the substantial downstaging efficacy of chemotherapy, using MRI after chemotherapy but before CRT. The FOWARC Chinese phase III study^43^ randomized 495 patients with LARC to either standard neoadjuvant CRT using concurrent 5‐FU, CRT with concurrent 5‐FU and oxaliplatin, or FOLFOX chemotherapy alone. Although tumour downstaging was comparable between the standard CRT and chemotherapy‐alone arms (37·1 and 35·5 per cent respectively), the pCR rate was inferior with chemotherapy alone (14·0 *versus* 6·6 per cent). It was reported recently that there was no difference in DFS or overall survival between the three arms^129^. At present, there is more evidence to support the replacement of neoadjuvant CRT with chemotherapy using DFS as the primary endpoint, than for a cCR/organ preservation endpoint.

## Discussion

The ideal radiosensitizing agent would be one that could target cancer cells selectively^130^, ^131^, enhancing the efficacy of treatment with minimal local and systemic toxicity. Exploiting the benefits of neoadjuvant therapy, accurately staging and assessing cCR could open up the era of increasingly personalized medicine and the avoidance of resection altogether^132^. It is an area of research that could bring significant patient benefits including improvements in health‐related quality of life. However, future clinical trials of radiosensitizers, with the aim of organ preservation, need carefully to consider the endpoints that are used to assess efficacy^132^.

Although there is much emerging evidence with regard to potential new radiosensitizing agents, the current standard treatment alongside RT remains 5‐FU or capecitabine chemotherapy. The addition of any second systemic agent has yet to show a consistent increase in efficacy in randomized studies. Many promising radiosensitizers have failed to progress beyond the preclinical and early clinical phases (I–II) owing to systemic toxicity and varying rates of pCR. Unfortunately, the quality of phase II studies of potential intensifying agents has been poor. A systematic review^133^ of 92 phase II trials showed that only eight were randomized.

There remains the fundamental question of the optimal primary endpoint. In virtually all studies in this review, the pCR rate was employed as the determinant of success. At present, there is no predetermined set definition of what constitutes a pCR. It may be defined as the absence of neoplastic cells in the surgical resection specimen as a result of neoadjuvant treatment (ypT0 and ypN0)^134^ and indeed may still occur even in the presence of mucosal abnormalities following treatment^135^. Published rates of pCR range widely from 15 to 40 per cent^136^, ^137^. However, despite small cohort sizes being accounted for, very few trials have noted the potential introduction of bias due to lack of standardization of pathologist reporting. The Royal College of Pathologists^138^ specifies that pathologists should embed all of the tumour‐associated scar and examine three deeper levels on each block before calling a pCR. The lack of reliable lymph node involvement status, dependence on pathologist block and level sampling intensity, and varying time points between the end of RT and surgery affecting tumour regression, could potentially lead to inflated pCR results. The associated benefits of a true pCR include a reduced recurrence rate and enhanced overall survival^136^, ^139^, ^140^.

Tumour regression grading is a semiquantitative assessment of residual tumour cells *versus* fibroinflammatory tissue in the rectal wall, and has been shown to be able to stratify tumour response to CRT and predict prognosis on an individual‐patient level in two large prospective phase III trials^141^, ^142^. Identifying patients who have achieved a cCR following CRT, and who could be followed prospectively with an active surveillance or watch‐and‐wait strategy, is gathering increasing interest. Of 183 patients with T2–T4 N0–2 M0 distal rectal cancers receiving neoadjuvant CRT in a trial published in 2014 by Habr‐Gama and colleagues^143^, 49 per cent were deemed to have achieved a cCR; 31 per cent of these patients went on to develop local recurrence and the salvage rate was 93 per cent. The rate of local disease control was 94 per cent, with 78 per cent organ preservation. The Habr‐Gama protocol involved clinical, endoscopic, radiological and serological reassessment of patients 8 weeks after completion of neoadjuvant therapy. A cCR was defined as the absence of residual ulceration, stenosis or mass lesion within the rectum on digital palpation and endoscopic imaging. MRI was performed, and the carcinoembryonic antigen level was measured.

The International Watch & Wait Database Consortium^144^ recently published the long‐term outcomes of the largest series of patients managed by this strategy, reporting a 2‐year cumulative regrowth rate of 25·2 per cent among 1009 patients. Surgical treatment data were available for only 148 of the 213 patients who experienced regrowth; 115 proceeded to TME resection, with histologically clear margins in 88 per cent. Overall 5‐year survival rates of 84·7 per cent in this group are comparable to those of major resection. However, before this approach can be established as a standard of care, standardized definitions of cCR and surveillance protocols need to be developed. Criteria for shared decision‐making with the patient for this approach also need to be addressed. An increasing consensus views a two‐stage assessment as optimum for identifying a cCR, at 3 and then 6 months following CRT, allowing enough time for a cCR to develop in initially good responders^145^. MRI tumour regression grade following CRT has been shown to be predictive of DFS in a cohort of 66 patients from the MERCURY study, suggesting the value of MRI assessment after CRT as part of the protocol for selecting patients for a non‐operative approach^146^. Although there are still many questions surrounding watch and wait^147^, it clearly has an increasingly important place in modern rectal cancer management and strategies to intensify CRT need further exploration.

For patients in whom there has been a good response but not an apparent cCR, local transanal excision is an alternative to major resection^148^, ^149^. MRI can be useful in guiding patient selection for such treatments^150^. However, the recently published GRECCAR 2 study^151^, which used a composite endpoint of surgical complications and recurrence, failed to show a difference between the two approaches in this setting, suggesting that more prospective studies are needed in this area.

It is imperative that studies employ standardized pathological reporting to ensure that the pCR rates quoted are both realistic and comparable. In view of this, use of the pCR as a primary endpoint for research studies and/or clinical trials should perhaps be questioned. If the ultimate goal is organ preservation regardless of whether the patient has undergone a cCR or pCR, perhaps organ preservation should be the primary endpoint. Against this is the morbidity associated with rectal surgery in terms of bowel, urinary and sexual dysfunction. However, there are few data on long‐term toxicity and health‐related quality of life for an active surveillance approach, which clearly needs to be addressed in future prospective studies. A recent patient consultation exercise revealed that, even in the context of cancer care, patients regarded quality of life and presence of a stoma as more important than overall survival^152^. Future trials of neoadjuvant therapy for rectal cancer need to ensure that patient experience and reported endpoints are addressed.

Although not available at the present time, it is hoped that the development of biomarker‐based stratified treatment will be used to guide neoadjuvant therapy on a personalized basis in the future^153^. Such biomarkers may be purely molecular (DNA alterations, gene expression, protein expression, epigenetic or circulating) or a combination of molecular markers and imaging findings^154^. Reliable pretreatment biomarkers do not currently exist, although ongoing research is attempting to identify pretreatment markers that are predictive of response^155^, ^156^. It is essential that future neoadjuvant trials incorporate a translational element to further develop biomarker‐guided therapy. As such, it is critical that such translational arms adhere to a robust biopsy protocol to ensure that enough appropriate biological material is available for downstream analysis in addition to the routine histopathological biopsies taken for diagnostic purposes. Factors that need to be considered include the person taking the biopsies, the quantity of material and timing. Patients who undergo a cCR or pCR will have little or no tumour to access at either clinical follow‐up or at the time of surgical resection; this must be considered in the translational design, which may need to include liquid biopsies^157^.
